# Sodium Monoiodoacetate Dose-Dependent Changes in Matrix Metalloproteinases and Inflammatory Components as Prognostic Factors for the Progression of Osteoarthritis

**DOI:** 10.3389/fphar.2021.643605

**Published:** 2021-04-28

**Authors:** Marta Bryk, Jakub Chwastek, Jakub Mlost, Magdalena Kostrzewa, Katarzyna Starowicz

**Affiliations:** Department of Neurochemistry, Maj Institute of Pharmacology, Polish Academy of Sciences, Cracow, Poland

**Keywords:** osteoarthritis, chronic pain, matrix metalloproteinases, cartilage, synovial membrane, synovial fluid, inflammation, pain

## Abstract

Osteoarthritis (OA) is a degenerative joint disease that primarily affects people over 65 years old. During OA progression irreversible cartilage, synovial membrane and subchondral bone degradation is observed, which results in the development of difficult-to-treat chronic pain. One of the most important factors in OA progression is joint inflammation. Both proinflammatory and anti-inflammatory factors, as well as extracellular matrix degradation enzymes (matrix metalloproteinases (MMPs), play an important role in disease development. One of the most widely used animal OA models involves an intra-articular injection of sodium monoiodoacetate (MIA) directly into the joint capsule, which results in glycolysis inhibition in chondrocytes and cartilage degeneration. This model mimics the degenerative changes observed in OA patients. However, the dose of MIA varies in the literature, ranging from 0.5 to 4.8 mg. The aim of our study was to characterize grading changes after injection of 1, 2 or 3 mg of MIA at the behavioral and molecular levels over a 28-day period. In the behavioral studies, MIA injection at all doses resulted in a gradual increase in tactile allodynia and resulted in abnormal weight bearing during free walking sequences. At several days post-OA induction, cartilage, synovial membrane and synovial fluid samples were collected, and qPCR and Western blot analyses were performed. We observed significant dose- and time-dependent changes in both gene expression and protein secretion levels. Inflammatory factors (CCL2, CXCL1, IL-1β, COMP) increased at the beginning of the experiment, indicating a transient inflammatory state connected to the MIA injection and, in more severe OA, also in the advanced stages of the disease. Overall, the results in the 1 mg MIA group were not consistently clear, indicating that the lowest tested dose may not be sufficient to induce long-lasting OA-like changes at the molecular level. In the 2 mg MIA group, significant alterations in the measured factors were observed. In the 3 mg MIA group, MMP-2, MMP-3, MMP-9, and MMP-13 levels showed very strong upregulation, which may cause overly strong reactions in animals. Therefore, a dose of 2 mg appears optimal, as it induces significant but not excessive OA-like changes in a rat model.

## Introduction

Knee osteoarthritis (OA) is one of the leading causes of global chronic disability. A disease that affects joint tissues, including cartilage, subchondral bone, ligaments, and muscles, OA manifests with chronic pain, joint stiffness/tenderness, loss of flexibility, and cracking sounds while moving. In the United States in 2016, 14 million people suffered from symptomatic knee OA. More than half of all patients were <65 years old (Deshpande et al., 2016). Risk factors favoring disease development include age, obesity, joint injuries, abnormal joint loading (e.g., excessive sports), gender (women are more likely to develop foot, hip and knee OA than men) and genetic factors (Vina and Kent Kwoh, 2018). Recently, the Osteoarthritis Research Society International (OARSI) revised the OA definition, adding an inflammatory component as a crucial factor contributing to disease development. An important process reported in OA progression is synovitis, during which a number of inflammatory changes occur in the synovial membrane. This contributes to local inflammation, which leads to further cartilage degeneration and inflammatory factor influx (Mathiessen and Conaghan, 2017).

Several of the most important factors are cytokines, both proinflammatory, e.g., interleukin 1 β (IL-1β), tumor necrosis factor α (TNFα), IL-6, IL-15, IL-17, and IL-18, and anti-inflammatory, such as IL-4, IL-10, and IL-13 (Wojdasiewicz et al., 2014). Importantly, a low-grade inflammatory state is present in OA from the very early stages, long before external symptoms can be detected by patients (Scanzello, 2017), and contributes to pain sensitization. Knee synovitis can also be used as an OA diagnostic tool (Berlinberg et al., 2019). Moreover, inflammation involves activation of enzymes involved in articular extracellular matrix (ECM) degradation – the matrix metalloproteinases (MMPs), e.g., MMP-2, MMP-3, MMP-9, MMP-13. These enzymes are members of a large family of zinc-dependent proteolytic enzymes. They are involved in shaping collagen, proteoglycan aggrecan, and non-collagen matrix components in joint degradation (Mehana et al., 2019). MMPs belong to one of three groups. MMP-2 and MMP-9 are gelatinases that digest denatured collagens and gelatins. MMP-2 (but not MMP-9) digests type I, II, and III collagens. MMP-2 in humans is important for osteogenesis, as mutations in human MMP-2 result in an autosomal recessive form of multicentric osteolysis (Martignetti et al., 2001). Both MMP-2 and MMP-9 are involved in inflammatory processes (Hannocks et al., 2019). MMP-3, also called Stromelysin 1, is responsible for digesting ECM components and activates a number of proMMPs (e.g., proMMP-1). MMP-13 is a collagenase that cleaves interstitial collagens type I, II, and III at a specific site three-quarters of the way from the N-terminus and digests both ECM and non-ECM components (Visse and Nagase, 2003).

Unfortunately, current OA therapeutics are limited to reducing pain and relieving bothersome symptoms only. A greater understanding of the mechanisms underlying the disease is necessary for creating more effective therapies. An important strategy for OA investigation is the creation of more representative animal models. Among several animal OA models, the sodium monoiodoacetate (MIA) intra-articular (i.a.) injection model is widely used for pain research and efficacy evaluation of therapeutic interventions (Lampropoulou-Adamidou et al., 2014). This chemical model mimics changes observed in patients very well. Malek et al. described changes in knee sensitivity and knee morphometric analysis in response to 3 mg of MIA i.a. injection up to 28 days post-injection (Malek et al., 2015). Knee changes 28 days post-MIA injection were irreversible and closely reflected changes in severe, advanced OA stages in human patients. In turn, Pajak et al. demonstrated biphasic pain progression in both behavioral and biochemical tests. An initial pain threshold lowering was associated with an inflammatory reaction caused by i.a. injection. Nevertheless, after 14 days, a stable, lasting reduction in pain threshold was observed (Pajak et al., 2017). The dose of MIA used to induce OA in rats varies in the literature from 0.5 to 4.8 mg; however, 1 or 3 mg is the most commonly used dose (Havelin et al., 2016; Allen et al., 2017; Haywood et al., 2018; Lee et al., 2018; Lockwood et al., 2019; Que et al., 2019; Piao et al., 2020). This could result in either peripheral mechanisms of joint damage in OA development and/or centrally mediated mechanisms, both of which may be important for future disease-modifying drug design. Therefore, the aim of our study was to assess the complete characteristics of grading MIA-induced OA (1, 2 or 3 mg of MIA i.a.) in a rat model at both the behavioral and molecular levels over a 28-day period. The role of inflammatory components and ECM MMPs in a rat joint model with progressive OA severity was investigated. Differences elicited by selected MIA doses were noted; our research allowed us to identify the smallest dose needed to effectively induce OA in a rat model. Overall, the obtained data allow for detailed description of changes occurring in joint tissues (synovial membrane, cartilage and synovial fluid) during disease progression, which provides a superior understanding of OA pathology and mechanisms.

## Materials and Methods

### Animals

In all experiments, male Wistar rats initially weighing 225–250 g were used (Charles River, Hamburg, Germany). Animals were housed 5 per cage in a 12/12 h light/dark cycle with food and water *ad libitum*. Behavioral experiments were performed between 9:00 and 12:00 am. Experiments were approved by the Local Bioethics Committee of the Maj Institute of Pharmacology (Cracow, Poland), approval numbers: 938/2012, 125/2018. Separate sets of animals were used for behavioral and biochemical studies. Depending on the experimental group, on days 2, 7, 21 or 28 of the experiment, animals were sacrificed, and joint tissue samples (cartilage, synovial membrane, synovial fluid) were collected. Healthy animals (examined via biochemical analysis) were sacrificed on various days (1 or 2 animals in each experimental day) to minimize the differences associated with the duration of the experiment. No differences in biochemical analysis were observed in the healthy group. In behavioral tests, animals were tested prior to MIA injection (day 0); to apply the 3R principle of animal testing, we determined that no healthy group was necessary. Moreover, according to the 3R principle and based on our previous behavioral research were 1 mg (Mlost et al., 2018b; Mugnaini et al., 2020; Mlost et al., 2021) or 3 mg of MIA (Malek et al., 2015; Pajak et al., 2017; Mlost et al., 2018a; Bryk et al., 2020) were successfully used, we decided to examine only 1 and 3 mg MIA doses in behavioral tests to minimize the number of animals exposed to painful procedures.

### Induction of OA

Animals were briefly anesthetized with 5% isoflurane (Aerrane, Baxter, United States) in 100% oxygen (3.5 L/min). The rear right knee skin surface was shaved and swabbed with 75% ethanol. Joint damage was induced by a single i.a. injection of 1, 2 or 3 mg of MIA (Sigma-Aldrich, Saint Louis, United States) dissolved in 50 µl of 0.9% physiological saline via a 30 G × 1/2” needle. All surgical procedures were performed under sterile conditions. After OA induction, animals were moved back into their home cages and observed until full recovery from anesthesia. Healthy animals did not undergo any injection. After i.a. injection, rats were maintained under the same conditions as the preoperative period. Changes in kinetic weight bearing (evaluated in the kinetic weight bearing test; KWB) and mechanical withdrawal thresholds (evaluated in von Frey’s test) were recorded prior to i.a. injection (day 0) and 2, 7, 14, 21 and 28 days post-injection. Experimental groups consisted of n = 5–8 animals (for KWB test), n = 8 animals (for Von Frey test) and n = 5–6 animals for biochemical studies. In rare cases, individual samples had to be excluded from the analysis due to abnormalities during isolation or sample contamination, and groups were indicated by an appropriate n number.

### Von Frey Test

For the assessment of mechanical allodynia, calibrated von Frey monofilaments (Bioseb, France) were used. Rats were placed in Plexiglas cages with a wire net floor 5 min before the experiment. Von Frey filaments were applied to the mid plantar surface of the ipsilateral hind paw according to the up and down method (Chaplan et al., 1994; Deuis et al., 2017). Each filament was applied three times for an approximately 2–3 s period or until a withdrawal response was evoked. After response, the paw was retested with monofilaments in descending order until no response occurred, at which point monofilaments were again applied in ascending order until the response could once again be evoked. The monofilament that evoked the final reflex was noted as the paw withdrawal latency. The strength of the von Frey monofilament bending forces was as follows: 0.4; 0.6; 1.0; 1.4; 2; 4; 6; 8; 10; 15 and 26 g as a cut-off for response.

### Kinetic Weight Bearing

To characterize pain behavior in the MIA model, KWB, a novel instrument developed by Bioseb (France), was used. Sensors placed on the ground measure the weight borne by each individual paw during the walking sequence of a freely moving animal, while a built-in camera detects the center of gravity of the animal. Data collection was terminated when 5 validated runs were obtained, or after 6 min of acquisition. If the animal did not run during this time window, the measurement was repeated at the end of the session. Those who failed to make at least one validated run during the second session were excluded from the analysis (one animal in 3 mg of MIA group in day 7 and 28). Rats were trained to move through the test corridor (50 × 130 cm) for a week before the actual experiment. Measurements were made directly before MIA administration and 2, 7, 21 and 28 days post-MIA treatment. All recorded data were validated by an observer blinded to the study. The final results include information about the mean peak force and surface area applied by each rear paw. The presented results discuss only the most relevant parameters in the context of OA research: peak force (centinewton, cN) – the mean of the maximum forces of each rear paw and peak surface (cm^2^) – the mean of the maximum surface of each rear paw.

### RNA and Protein Isolation

Cartilage from the medial femoral condyle and synovial membrane fragments were collected with surgical scissors. After tissue collection, each sample was placed in RNA-later solution (Invitrogen, United States) and stored at −80°C. RNA and protein from the synovial membrane were isolated using TRIzol Reagent (Invitrogen, United States) according to the manufacturer's protocol. Extraction of high-quality RNA from cartilage was performed according to a protocol published by Le Bleu et al. (2017). Synovial fluid was collected by rinsing the joint capsule with 50 μl of physiological saline and aspiring the fluid with a syringe with a 25 G × 5/8” needle. Then, synovial fluid was immediately placed on ice and frozen at −80 °C. Equal volumes of samples were lyzed with 2% SDS and centrifuged at 12,000 × g. The supernatant was collected for immunoblotting.

Total RNA levels were measured with a Nanodrop spectrophotometer (ND-1000, Nanodrop; Labtech International, United Kingdom). Each sample was equalized to a concentration of 1 μg/μl and reverse transcribed to cDNA using an NG dART RT kit (EURx, Poland) according to the manufacturer’s protocol. qPCR reactions were carried out using iTaq Universal Probes Supermix (Bio-Rad, United States) and TaqMan Assays (Thermo Fisher, Applied Biosystems, United States) in a Thermal Cycler CFX96 (Bio-Rad, United States). Cycle threshold values were calculated automatically via CFX Manager software. RNA abundance was calculated as ddCT 2^-(threshold cycle)^ and *B2m* normalized. The TaqMan assay protocols used in the study are presented in the Supplementary Material.

### Western Blot

The protein levels in the cartilage and synovial samples were estimated via BCA kit (Thermo-Fischer, United States), and equal amounts of proteins were denatured in 4 × Laemmli sample buffer (Bio-Rad, United States) and denatured at 96°C for 6 min. Equal volumes of synovial fluid samples were lyzed with 2% SDS and denatured with Laemmli buffer, such as cartilage and synovial samples. An equal amount of protein from the various experimental groups was separated on Criterion TGX 4–20% precast gels (Bio-Rad, United States) and transferred onto a PVDF membrane (Roche, Switzerland). After blocking with blocking reagent from the BM Chemiluminescence Western Blotting Kit (Roche, Switzerland), membranes were incubated overnight with primary antibodies (detailed information in the Supplementary Material) using a SignalBoost Immunoreaction Enhancer Kit (Merc, Germany). The amount of β-actin was measured on the same membrane on which the other proteins were measured by mild stripping (using a protocol published by Abcam). In control samples of synovial fluid concentration of proteins was very low, so this tissue was normalized to volume and detection of reference proteins was not possible.

### Statistical Analysis

Statistical analysis was performed using Statistica 13 (SatSoft Software, Tulsa, Oklahoma, Unitd States), and graphs were generated using Prism 8 (GraphPad Software La Jolla, California, United tates). Data are presented as the mean ± SEM; whiskers on the boxplot graphs show the minimum and maximum values. The results of the von Frey test were evaluated by two-way analysis of variance (ANOVA), followed by Tukey’s HSD post-hoc test. The results from the KWB test were evaluated by one-way ANOVA on day 0 or two-way ANOVA on the following experimental days, followed by Tukey’s HSD post-hoc test. The qPCR and Western blot results were evaluated by one-way ANOVA followed by Dunnett’s post-hoc test, treating healthy animals as the control group. In behavioral tests, each group consisted of eight animals (von Frey test) or 5 animals (KWB test). In biochemical analyses, the experimental group sizes consisted of 3-6 samples per group (qPCR) or 4-5 samples per group (Western blot). Values of *p* < 0.05 (denoted * or #), *p* < 0.01 (denoted ** or ##), and *p* < 0.001 (denoted *** or ###) were considered to be statistically significant.

## Results

A significant difference in pain threshold was observed in both MIA doses, using the von Frey and KWB tests. Differences in tactile allodynia, as well as in paw pressure and surfaces applied to the ground between the rear right and rear left paws on subsequent experimental days were observed. Significant signs of pain were noticed from the 2nd day post-MIA treatment and persisted until the day 28. In biochemical analyses, statistically significant dose- and time-dependent changes in MMP and inflammation-related gene expression (qPCR) and protein production (Western blot technique) were observed. The results will be explained in details in the following sections.

### Development of OA-Related Allodynia

**FIGURE 1:**
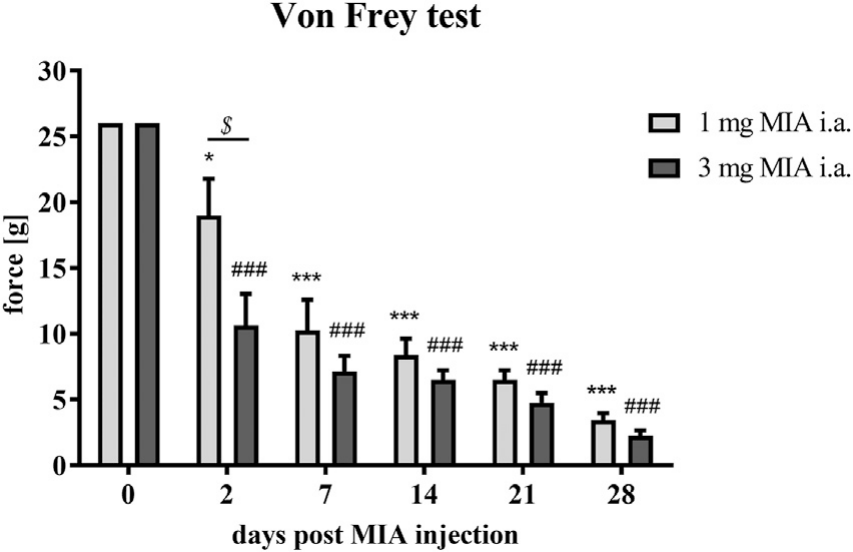
Mechanical allodynia measured by quantifying tactile sensitivity during OA developme with MIA grading. Pain sensitivity was measured with von Frey filaments in the ipsilateral paw (rear right) prior to MIA injection and 2, 7, 14, 21, and 28 days post-MIA treatment (1 or 3 mg intra-articular injection; i. a.; n = 8 animals per group). Two-way ANOVA and Tukey’s HSD post-hoc testing was performed. * denotes *p* < 0.05; *** denotes *p* < 0.001 in each day vs. day 0 in the 1 mg group; ### denotes *p* < 0.001 in each day vs. day 0 in the 3 mg group, $ denotes *p* < 0.05 1 mg vs. the 3 mg group.

MIA injection at both investigated doses (1 and 3 mg MIA i.a.) resulted in the development of mechanical allodynia in the ipsilateral hind paw (rear right) on day 2 (*p <* 0.05 for 1 mg of MIA and *p* < 0.001 for 3 mg of MIA; Figure 1), a phenomenon not observed on day 0. On day 2 a significant difference between tested doses was also observed (*p <* 0.05; Figure 1), which was not observed in the following days. OA rats showed a significant gradual reduction in withdrawal threshold that progressed with time in both experimental groups. The lowest mechanical withdrawal latency was observed at day 28. From day 7 on, the effect was similar in both groups (1 and 3 mg of MIA; *p <* 0.001*;* Figure 1). These results indicate that animals in whom OA symptoms were induced with both MIA doses, developed an OA-associated allodynia.

### Paw Weight Bearing Differences During Free Walk

**FIGURE 2:**
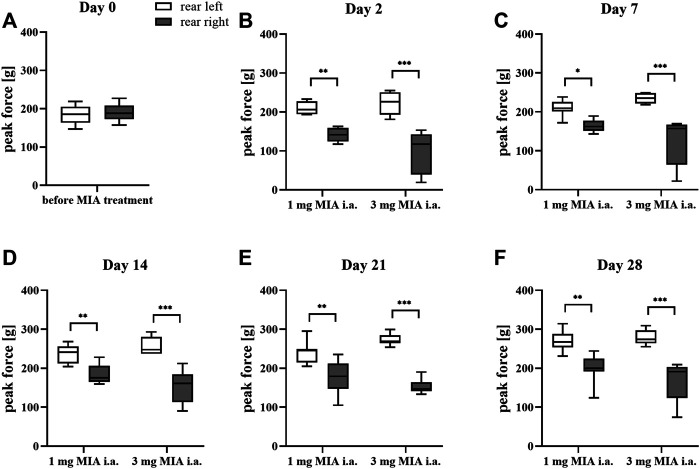
Gait analysis in OA rats recorded with KWB by measuring the distribution of paw force applied to the ground during a free walk (for 1 and 3 mg of MIA i.a. groups) during a free walk. Measurements taken prior to the MIA injection (day 0; **A**) and 2, 7, 14, 21, and 28 days **(B–F)** post-MIA treatment (n = 5–8 animals per group). One-way ANOVA on day 0 or two-way ANOVA on the following experimental days and Tukey’s HSD post-hoc tests were performed. * denotes *p* < 0.05; ** denotes *p* < 0.01; *** denotes *p* < 0.001.

**FIGURE 3:**
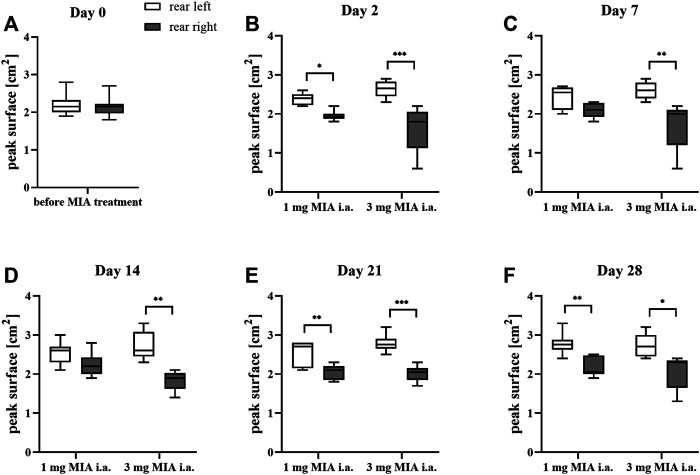
Gait analysis in OA rats recorded with KWB by measuring the distribution of paw surfaces applied to the ground during free walks (for 1 and 3 mg of MIA i.a. groups) during a free walk. Measurements taken prior to the MIA injection (day 0; **A**) and 2, 7, 14, 21, and 28 days **(B–F)** post MIA treatment (n = 5–8 animals per group). One-way ANOVA on day 0 or two-way ANOVA on the following experimental days and Tukey’s HSD post hoc test were performed. * denotes *p* < 0.05; ** denotes *p* < 0.01; *** denotes *p* < 0.001.

Rats were evaluated for paw force and surface distribution during a free walking sequence. A significant difference in both paw force (Figures 2A–F) and surface (Figures 3A–F) applied to the ground during free walking was observed for both MIA doses used to induce OA. Differences were observed in both experimental groups (1 or 3 mg MIA i.a.) on almost all experimental days (except days 7 and 14 in the peak surface analysis of the 1 mg MIA group); however, a stronger effect was elicited by 3 mg of MIA. Therefore it can be concluded that both MIA doses induced weight bearing differences in MIA-treated rats.

### Inflammation During OA Progression

**FIGURE 4:**
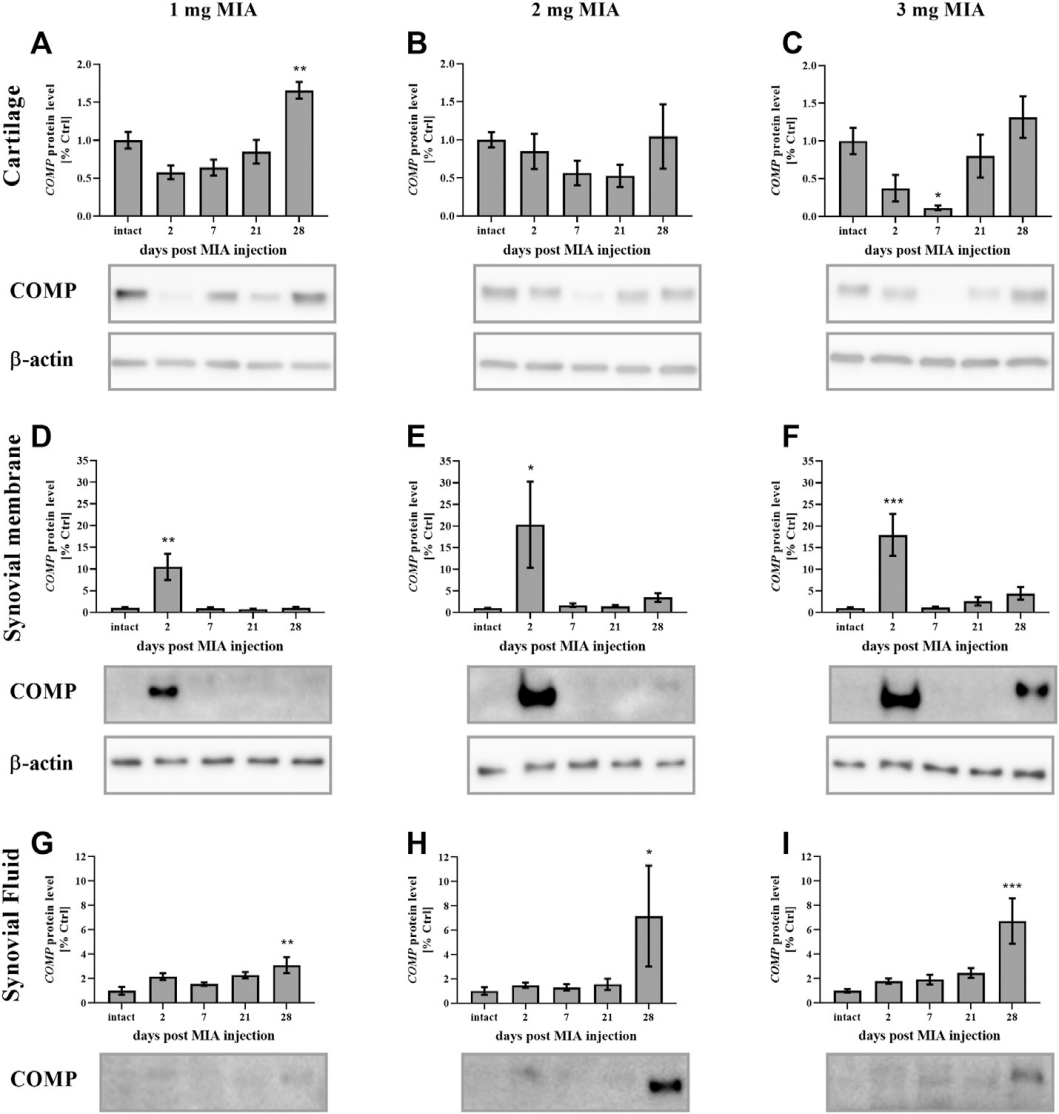
Changes in COMP protein levels in cartilage **(A–C)**, synovial membrane **(D–F)** and synovial fluid **(G–I)** samples from osteoarthritic rats as measured by Western blot assay. Tissues were collected 2, 7, 21 or 28 days after OA induction. Results are presented as mean group fold change ± SEM in comparison to the control group (healthy animals), n = 4–5 samples per group. Data were analyzed with one-way ANOVA followed by Dunnett’s post-hoc test. * denotes *p* < 0.05; ** denotes *p* < 0.01; *** denotes *p* < 0.001 vs. intact animals.

**FIGURE 5:**
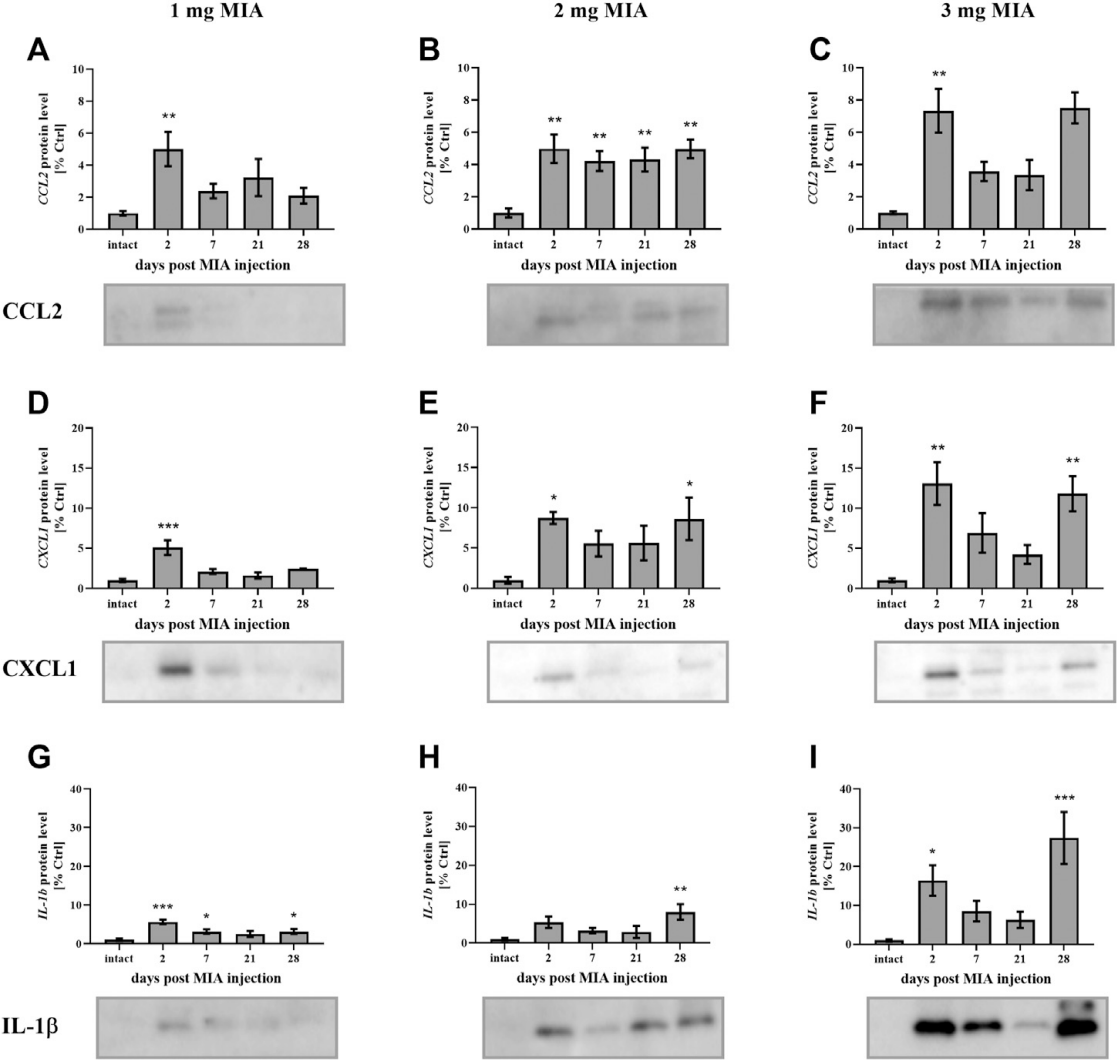
Changes in the protein levels of the inflammation-related factors CCL2 **(A–C)**, CXCL1 **(D–F)**, and IL-1β **(G–I)** in the synovial fluid of osteoarthritic rats, as measured by Western blot assay. Tissues were collected 2, 7, 21 or 28 days after OA induction. The results are presented as the mean group fold change ± SEM in comparison to the control group (healthy animals), n = 5–6 samples per group. Data were analyzed with one-way ANOVA followed by Dunnett’s post hoc test. * denotes *p* < 0.05; ** denotes *p* < 0.01; *** denotes *p* < 0.001 vs. intact animals.

In the present study, cartilage oligomeric matrix protein (COMP) levels were measured in joint tissues isolated from osteoarthritic animals. In cartilage samples, a U-shaped pattern of protein production was observed following injection, with an initial decrease (significant only on day 7 in the 3 mg MIA group; *p* < 0.05; Figure 4C) and subsequent return to baseline (in the 2 and 3 mg MIA groups) or an increase (in the 1 mg MIA group; 0.01 > *p >* 0.001; Figures 4A–C). In synovial membranes, in contrast, an initial increase (day 2) was solely observed in all experimental groups (MIA 1 mg; 0.01 *> p >* 0.001; MIA 2 mg; *p <* 0.05; MIA 3 mg; *p* < 0.001; Figures 4D–F). In synovial fluid samples, an increase in COMP levels was detected in the latter stages of OA – exclusively on day 28 post-MIA treatment – in all groups (MIA 1 mg; 0.01 > *p* > 0.001; MIA 2 mg; *p <* 0.05; MIA 3 mg; *p* < 0.001; Figures 4G–I). The levels of various inflammation-related factors were measured only in the synovial fluid. Chemokine (C-C motif) ligand 2 (CCL2) levels showed an initial increase (day 2 at all MIA doses; 0.01 *> p >* 0.001; Figures 5A–C). In the 1 mg MIA group, the CCL2 level returned to baseline, whereas in the 2 mg MIA group, it remained elevated throughout the experiment (0.01 *< p <* 0.001; Figures 5A,B). In the 3 mg MIA group, the CCL2 level was increased throughout the entire experimental period; however, these differences reached statistical significance only on day 2 (0.01 *> p >* 0.001; Figure 5C). A similar pattern was observed for chemokine (C-X-C motif) ligand 1 (CXCL1). The protein level was increased in synovial fluid by the 2^nd^ day post-OA induction in all groups (MIA 1 mg; *p <* 0.001; MIA 2 mg; *p <* 0.05; MIA 3 mg; 0.01 > *p <* 0.001; Figures 5D–F). In the 1 mg MIA group, this increase returned to baseline, whereas in the 2 and 3 mg MIA groups, it remained significantly upregulated on day 28 post-MIA treatment (MIA 2 mg; *p* < 0.05; MIA 3 mg; 0.01 *> p >* 0.001; Figures 5D–F). The IL-1β levels in the synovial fluid in the 1 mg MIA group were increased on days 2 (*p <* 0.001), 7 (*p* < 0.05) and 28 (*p* < 0.05) post-MIA treatment (Figure 5G). In the 2 mg MIA group, this expression level was increased only on day 28 post-MIA treatment (0.01 *> p >* 0.001; Figure 5H). At the highest MIA dose (3 mg), IL-1β levels were increased on days 2 (*p* < 0.05) and 28 (*p <* 0.001) post-OA induction (Figure 5I). The above results show a dose-dependent inflammation in joint tissues isolated from osteoarthritic animals. The strongest effect was observed in the group in which OA was induced with MIA at a dose of 3 mg, however, a comparable effect was triggered by 2 mg of MIA.

### Matrix Metalloproteinase Expression Changes During OA Progression

#### Changes in MMP Gene Expression During OA Development

MMP gene expression was measured in synovial membrane and cartilage tissues of osteoarthritic rats. *Mmp-2* gene expression significantly increased in the later stages of OA. In the synovial membrane samples, an increase was observed 7 days post-MIA injection (at all MIA doses; MIA 1 mg; 0.01 *> p* > 0.001; MIA 2 mg; *p <* 0.001; MIA 3 mg; *p <* 0.001*;* Table 1), 21 days post-MIA injection (2 and 3 mg MIA groups; 0.01 *< p <* 0.001 and *p <* 0.001 respectively) and 28 days post-MIA injection (1 and 3 mg MIA groups 0.01*<p <* 0.001 and *p* < 0.001 respectively). In the 2 mg MIA group, the *Mmp-2* expression level remained elevated on day 28 (similar to the 1 mg MIA group); however, these differences did not reach statistical significance. Similar results were observed in cartilage samples—an increase in *Mmp-2* abundance was observed starting at day 7 (in the 2 mg MIA group; *p* < 0.05) or day 21 (in the 1 and 3 mg MIA groups; 0.01 *> p* > 0.001 and *p <* 0.001 respectively) that persisted until the end of the experiment (MIA 1 mg; *p <* 0.05; MIA 2 mg; 0.01 > *p >* 0.001; MIA 3 mg; *p* < 0.001). The *Mmp-3* expression level in the 1 mg MIA group was elevated in the initial stages of OA (days 2 and 7; 0.01 *> p >* 0.001; in the synovial membranes or day 2 in cartilage; *p <* 0.05). In the synovial membranes of the 2 mg MIA group, an increase in *Mmp-3* gene expression was observed starting at the beginning of the experiment; however, significance was reached only on day 21 (*p <* 0.05). In the cartilage samples, significant upregulation was observed on days 7 (*p <* 0.05) and 28 (*p* < 0.001) post-MIA treatment. In the synovial membranes samples of the 3 mg MIA group, *Mmp-3* expression was elevated throughout the entire experimental period (day 2; 7; 21; 0.01 *> p >* 0.001; day 28 *p <* 0.001); in cartilage, it was elevated only in the later stages (days 21 and 28; 0.01 *> p >* 0.001 and *p* < 0.001 respectively). The *Mmp-9* expression level in the synovial membranes was upregulated in the 1 mg MIA group, with a significant increase on days 2 (*p <* 0.05), 7 (*p* < 0.001) and 28 (*p* < 0.001) post-MIA treatment. In the 2 mg MIA group, the *Mmp-9* level increased; however, group variances were too substantial to reach statistical significance. In the 3 mg MIA group, *Mmp-9* expression also increased throughout the entire experimental period, with a significant elevation observed on experimental days 7 (*p* < 0.001) and 21 (0.01 *> p >* 0.001). In the cartilage samples, a significant increase in *Mmp-9* levels was observed at the advanced OA stages (on day 21in the 1 mg MIA group; 0.01 *> p >* 0.001, on day 28 in the 2 mg MIA group; *p* < 0.05; and on days 7 and 21 in the 3 mg MIA group; *p <* 0.05). Nevertheless, a trend toward increasing expression was observed throughout the entire experiment, particularly in the synovial membrane samples. For *Mmp-13* gene expression, in the 1 mg MIA group, an elevation was observed only at the beginning of the experiment (day 2) in both tissue types (Synovial membrane; *p <* 0.001*;* Cartilage; *p <* 0.05). In the 2 mg MIA group, no results reached statistical significance. In the 3 mg MIA group, in the synovial membrane, an increase was observed throughout nearly the entire experiment (days 7, 21 and 28; *p <* 0.05); in cartilage, elevated expression of Mmp-13 was observed solely on day 21 post-MIA treatment (0.01 *> p >* 0.001). The results of this gene expression analysis are shown in Table 1. *Comp* expression in cartilage showed a decrease in the initial phases of OA in the 1 mg MIA group (day 2 and 7; 0.01 *> p >* 0.001) and a subsequent increase in the 2 (*p <* 0.05) and 3 mg (0.01 *> p >* 0.001) MIA groups on day 21 (see Supplementary Table S1). In summary, the qPCR test showed a dose- and time-dependent changes in mRNA levels of *Mmps*.

**TABLE 1:** Transcript abundance levels of selected genes in synovial membrane and cartilage samples from osteoarthritic rats.

Tissue	Gene	MIA [mg]	Days post-MIA injection				
			Ctrl	2	7	21	28
Synovial membrane	*Mmp-2*	1	1.1 ± 0.2	1.7 ± 0.4	2,9** ± 0,5	1.7 ± 0.1	2,6** ± 0,1
		2		.9 ± 0.1	6,4*** ± 0,8	4,1** ± 1,0	2.5 ± 0.9
		3		1.1 ± 0.1	7,2*** ± 0,5	3,5*** ± 0,3	4,4*** ± 0,3
	*Mmp-3*	1	1.0 ± 0.1	6,4** ± 2,1	9,8** ± 2,0	2.3 ± 0.2	2.5 ± 0.6
		2		7.0 ± 1.1	5.9 ± 1.0	10,7* ± 5,3	2.9 ± 0.4
		3		7,3** ± 1,6	6,7** ± 1,2	7,4** ± 0,9	14,0*** ± 1,9
	*Mmp-9*	1	1,1 ± 0,2	9,6* ± 1,6	30,7*** ± 1,8	4,6 ± 2,1	35,8*** ± 4,9
		2		27.9 ± 4.4	26.5 ± 6.0	40.4 ± 15,4	36.9 ± 33.4
		3		28.6 ± 4.6	94,5*** ± 18,3	75,1** ± 25,8	20.6 ± 1.8
	*Mmp-13*	1	1,5 ± 0,7	179,2*** ± 55,6	53,2 ± 11,9	3,1 ± 1,3	3,2 ± 0,9
		2		163.8 ± 67.9	42.1 ± 5.2	137.1 ± 103.4	12.0 ± 5.4
		3		53.3 ± 17.2	65,0* ± 9,5	80,5* ± 28,8	67,8* ± 24,3
Cartilage	*Mmp-2*	1	1.1 ± 0.3	2.3 ± 0.7	1.8 ± 0.7	4,6** ± 1,1	3,9* ± 0,8
		2		1.1 ± 0.3	2,7* ± 0,8	3,3** ± 0,5	3,5** ± 0,5
		3		1.3 ± 0.2	1.1 ± 0.4	8,1*** ± 0,8	4,3*** ± 0,7
	*Mmp-3*	1	1.1 ± 0.2	4,4* ± 1,3	2.8 ± 0.4	2.2 ± 0.9	1.7 ± 0.6
		2		2.2 ± 0.7	3,5* ± 0,8	2.3 ± 0.7	5,2*** ± 1,0
		3		2.3 ± 0.8	2.2 ± 0.3	4,2** ± 1,0	5,5*** ± 0,8
	*Mmp-9*	1	1.0 ± 0.1	0.9 ± 0.1	1.1 ± 0.1	1,9** ± 0,3	1.3 ± 0.2
		2		1.1 ± 0.1	1.5 ± 0.2	1.6 ± 0.2	2,6* ± 0,9
		3		1.1 ± 0.1	1,8* ± 0,2	1,8* ± 0,3	1.6 ± 0.2
	*Mmp-13*	1	1.2 ± 0.3	2,7* ± 0,5	0.9 ± 0.2	1.1 ± 0.3	1.8 ± 0.7
		2		1.1 ± 0.3	0.8 ± 0.2	1.2 ± 0.2	1.3 ± 0.5
		3		1.4 ± 0.3	0.6 ± 0.3	2,7** ± 0,4	1.1 ± 0.3

The results were assessed by quantitative PCR (qPCR). Total RNA samples were collected 2, 7, 21 or 28 days after OA induction. The results are presented as the mean group fold change ± SEM in comparison to the control group (healthy animals), n = 3–6 samples per group. Data were analyzed with one-way ANOVA followed by Dunnett’s post-hoc test.

^* ^denotes p < 0.05.

^**^ denotes p < 0.01.

^***^ denotes p < 0.001 vs. intact animals (Ctrl).

Statistically significant values are shown in bold.

#### MMP Protein Levels During OA Development

**FIGURE 6:**
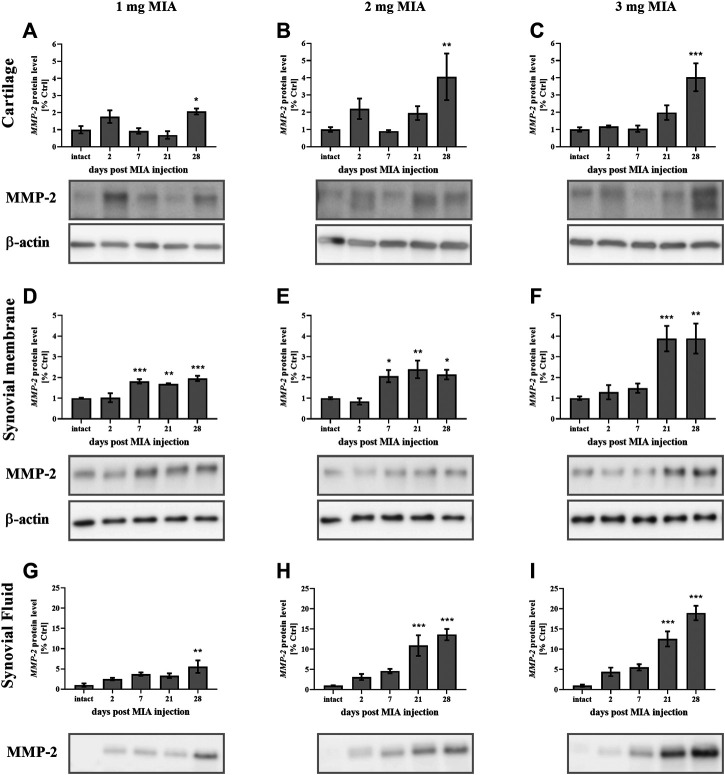
Changes in MMP-2 protein levels in cartilage **(A–C)**, synovial membrane **(D–F)** and synovial fluid **(G–I)** samples of osteoarthritic rats as measured by Western blot assay. Tissues were collected 2, 7, 21 or 28 days after OA induction. The results are presented as the mean group fold change ± SEM in comparison to the control group (healthy animals), n = 4–5 samples per group. Data were analyzed with one-way ANOVA followed by Dunnett’s post-hoc test. * denotes *p* < 0.05; ** denotes *p* < 0.01; *** denotes *p* < 0.001 vs. intact animals.

MMP-2 protein secretion was elevated in advanced OA stages in all experimental groups. In cartilage samples, a significant increase was observed solely on day 28 of the experiment at all MIA doses (MIA 1 mg; *p* < 0.05; MIA 2 mg; 0.01 *> p >* 0.001; MIA 3 mg; *p* < 0.001*;* Figures 6A–C). In synovial membrane samples, MMP-2 protein levels were increased starting on day 7 (1 and 2 mg MIA groups; *p* < 0.001 and *p* < 0.05) or day 21 (3 mg MIA group; *p* < 0.001) and remained elevated until the end of the experiment (Figures 6D–F). In the synovial fluid of OA rats, MMP-2 was increased at the later stages of the disease, on day 28 (1 mg MIA group; 0.01 *> p >* 0.001) or days 21 and 28 (2 and 3 mg MIA groups; *p <* 0.001; Figures 6G–I).

**FIGURE 7:**
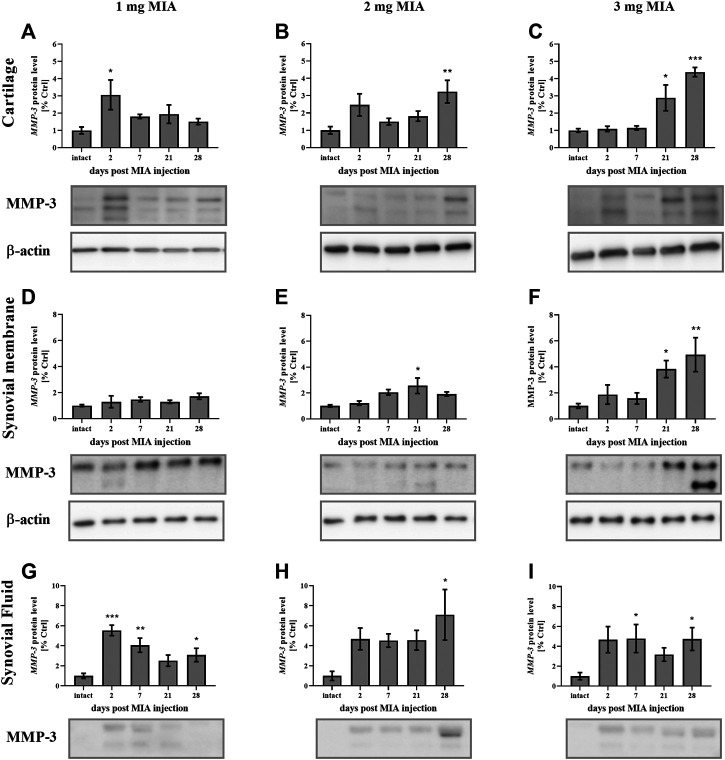
Changes in MMP-3 protein levels in cartilage **(A–C)**, synovial membrane **(D–F)** and synovial fluid **(G–I)** samples of osteoarthritic rats as measured by Western blot assay. Tissues were collected 2, 7, 21 or 28 days after OA induction. The results are presented as the mean group fold change ± SEM in comparison to the control group (healthy animals), n = 4–5 samples per group. Data were analyzed with one-way ANOVA followed by Dunnett’s post-hoc test. * denotes *p* < 0.05; ** denotes *p* < 0.01; *** denotes *p* < 0.001 vs. intact animals.

MMP-3 protein levels, in cartilage samples in the 1 mg MIA group, showed an early increase (on day 2 post-MIA treatment; *p* < 0.05*;* Figure 7A). A similar trend was observed in the 2 mg MIA group; however, these results did not reach statistical significance. In the 2 and 3 mg MIA groups, a significant increase in MMP-3 levels was observed in the later OA stages (days 28; 0.01 *> p >* 0.001; or days 21;*p* < 0.05; and 28; *p <* 0.001; respectively; Figures 7B,C). In the synovial membrane samples, the lowest MIA dose did not significantly alter MMP-3 protein production (Figure 7D). In the 2 mg MIA group, an increase was observed only on day 21 (*p* < 0.05), whereas in the 3 mg MIA group, a significant increase was observed on days 21 and 28 post-MIA injection (*p <* 0.05 and 0.01 *> p >* 0.001*;* Figures 7E,F).

In the synovial fluid samples, an increase in MMP-3 protein levels was detected in the early stages of OA. The lowest dose of MIA (1 mg) resulted in an early release of MMP-3 into the synovial fluid; a significant elevation was observed on days 2, 7 and 28, with a peak on day 2 (*p <* 0.001 and 0.01 *> p >* 0.001*; p <* 0.05 respectively; Figure 7G). In the 2 mg MIA group, a trend toward increased expression was observed from the beginning of the experiment; however, only the results on day 28 reached statistical significance (*p <* 0.05*;* Figure 7H). Similar results were observed in the 3 mg MIA group, with a significant increase in MMP-3 levels on days 7 and 28 (*p <* 0.05*;* Figure 7I).

**FIGURE 8:**
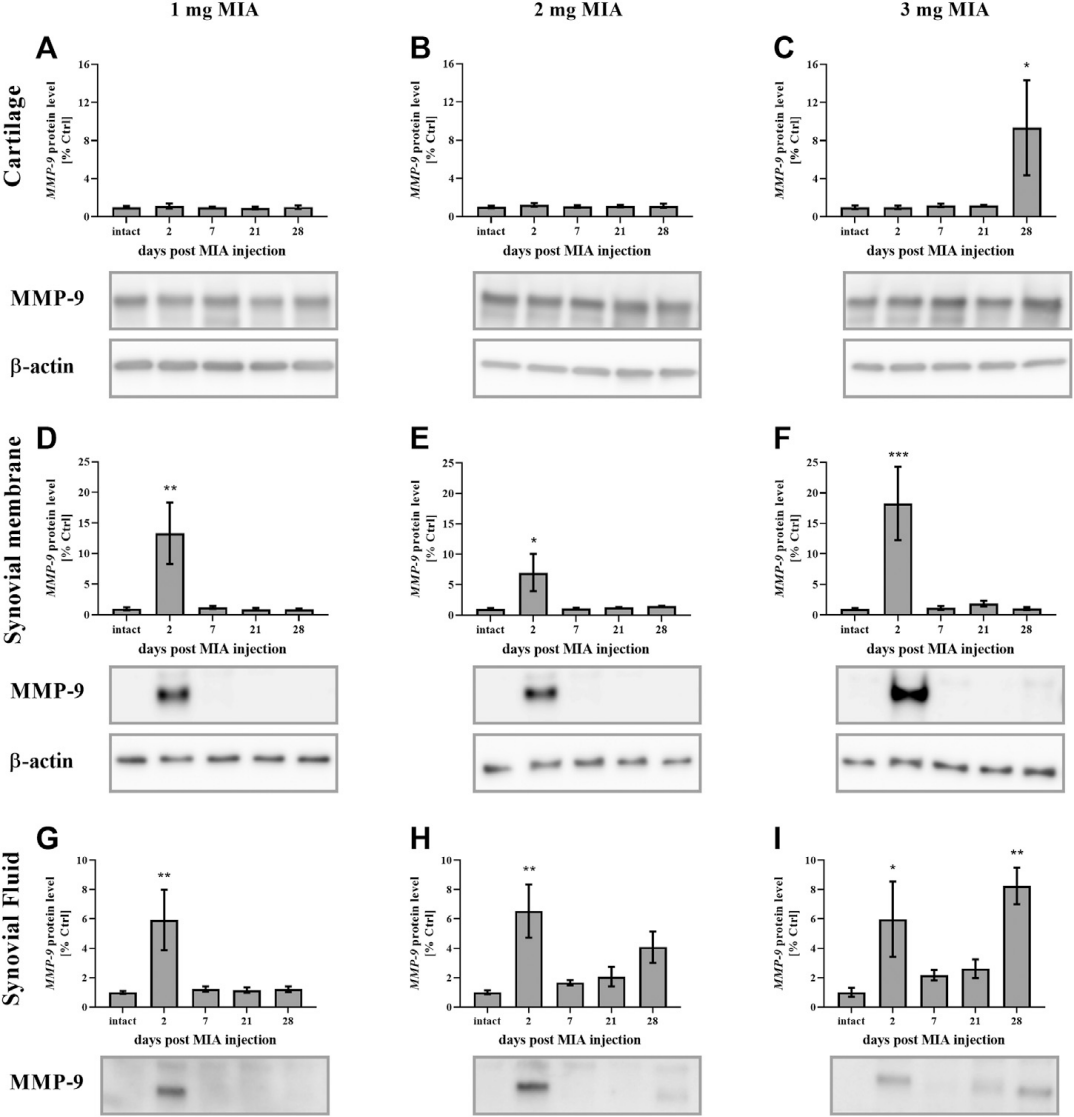
Changes in MMP-9 protein levels in cartilage **(A–C)**, synovial membrane **(D–F)** and synovial fluid **(G–I)** samples of osteoarthritic rats as measured by Western blot assay. Tissues were collected 2, 7, 21 or 28 days after OA induction. The results are presented as the mean group fold change ± SEM in comparison to the control group (healthy animals), n = 4–5 samples per group. Data were analyzed with one-way ANOVA followed by Dunnett’s post-hoc test. * denotes *p* < 0.05; ** denotes *p* < 0.01; *** denotes *p* < 0.001 vs. intact animals.

MMP-9 protein levels in cartilage samples at low MIA doses (1 and 2 mg MIA groups) showed no significant changes (Figures 8A,B). However, in the 3 mg MIA group, MMP-9 protein levels increased significantly on day 28 (*p* < 0.05*;* Figure 8C). In synovial membrane samples, in all tested MIA doses, MMP-9 levels increased exclusively in the early OA stages (day 2 post MIA treatment; MIA 1 mg; 0.01 *> p* > 0.001; MIA 2 mg; *p <* 0.05; MIA 3 mg; *p* < 0.001), subsequently returning to baseline in the following experimental days (Figures 8D–F). Similar observations were noted for the synovial fluid samples – an early (day 2) increase in MMP-9 protein levels was detected at all MIA doses (MIA 1 mg; 0.01 *> p >* 0.001; MIA 2 mg; 0.01 > *p >* 0.001; MIA 3 mg; *p* < 0.05), followed by a return nearly to baseline and a final increase on the last experimental day (a significant increase was observed only in the 3 mg MIA group (0.01 *> p* > 0.001). However, a trend toward increased expression was also observed in the 2 mg MIA group; Figures 8G–I).

**FIGURE 9:**
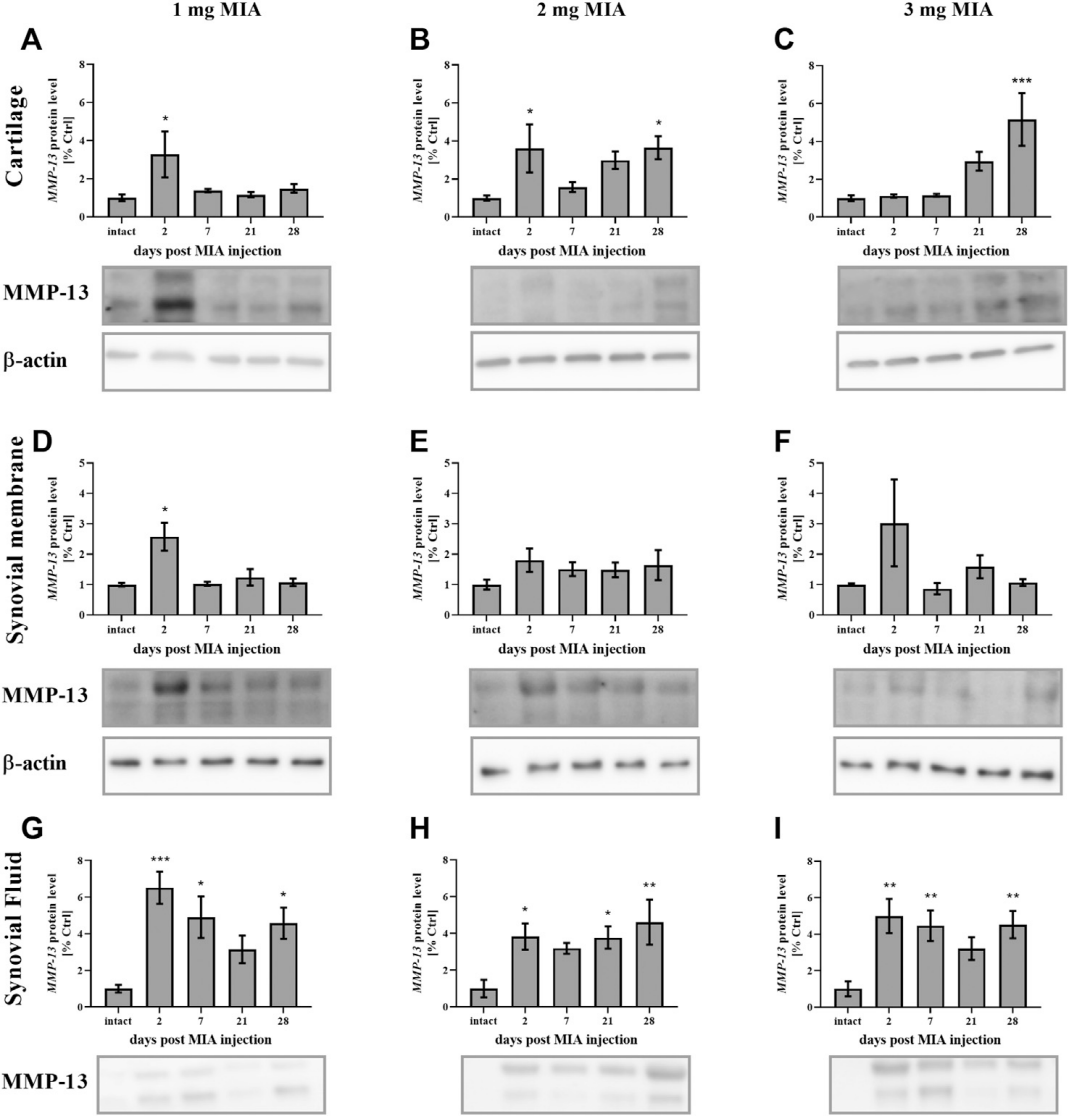
Changes in MMP-13 protein levels in cartilage **(A–C)**, synovial membrane **(D–F)** and synovial fluid **(G–I)** samples of osteoarthritic rats as measured by Western blot assay. Tissues were collected 2, 7, 21 or 28 days after OA induction. The results are presented as the mean group fold change ± SEM in comparison to the control group (healthy animals), n = 4–5 samples per group. Data were analyzed with one-way ANOVA followed by Dunnett’s post-hoc test. * denotes *p* < 0.05; ** denotes *p* < 0.01; *** denotes *p* < 0.001 vs. intact animals.

Regarding MMP-13, lower MIA doses (1 and 2 mg) were associated with an early increase (day 2) in MMP-13 production in cartilage (*p <* 0.05*;* both groups). However, in the 2 mg MIA group, an increase was also observed in the later OA stages (day 28; *p <* 0.05, with a trend toward increased expression on day 21; Figures 9A,B). In the 3 mg MIA group, there was a robust increase in MMP-13 levels on day 28 (*p <* 0.001; Figure 9C). In the synovial membrane, ambiguous results were obtained, with a significant increase identified only on day 2 in the 1 mg MIA group (*p* < 0.05; Figures 9D–F). In the synovial fluid samples, an increase in Mmp-13 levels was observed for almost the entire experimental period. In the 1 and 3 mg MIA groups, a significant increase was detected on days 2, 7 and 28 post-MIA treatment, whereas in the 2 mg MIA group, Mmp-13 levels significantly increased on days 2, 21 and 28 (Figures 9G–I).

In summary, protein levels for investigated MMPs changed significantly in a dose- and time-dependent manner. All MIA doses significantly changed protein concentration in the synovial fluid, whereas in cartilage and synovial membrane significant changes were observed mostly in 2 and 3 mg of MIA groups.

## Discussion

In the current study, behavioral and biochemical OA-related changes were investigated. The molecular alterations in MMP and inflammation-related factor expression were measured during 28 days of OA progression across three MIA dose groups: 1, 2 or 3 mg of MIA i.a. In the behavioral study, both investigated MIA doses (1 and 3 mg) caused significant allodynia and disturbed weight bearing patterns starting on the second day of the experiment. Although a stronger effect was observed in the 3 mg MIA group, both doses were sufficient to trigger OA-like pain behavior in rats. Furthermore, at the molecular level, significant differences between the investigated doses were observed.

OA progression is associated with an inflammatory state (Goldring and Otero, 2011). OA-related knee pain correlates with joint synovitis, subarticular bone attrition, bone marrow lesions and meniscal tears (Torres et al., 2006; Baker et al., 2010). Moreover, synovitis and effusion are associated with cartilage degeneration and loss in human patients (Roemer et al., 2011). There are several pathways that reportedly play an important role in synovitis progression. Activated macrophages promote catabolic mediator production and Toll-like receptor (TLR) and NFκB pathway activation, which leads to proinflammatory chemokine and cytokine production (e.g., IL-1β, IL-2, IL-6, IL-8, IL-15, TNFα, CCL2, CCL5, CCL19) (Scanzello and Goldring, 2012). Malek et al. previously observed an early increase in pain-related behavior in MIA-treated rats associated with transient inflammation triggered by i.a. MIA injection (Malek et al., 2015). Here, we observed an early peak (on day 2 post-MIA injection) in the gene expression of inflammation-related factors (*Ccl2, Cxcl-1, and Il-6*) in synovial membrane samples across all MIA doses (see Supplementary Table S1) and in protein levels in synovial fluid (CCL2, CXCL1, and IL-1β).

One of the most promising molecular markers of OA is COMP, which regulates and stabilizes the collagen network in cartilaginous tissue (Živanović et al., 2011). Upregulation of this protein in OA patient serum has been correlated with disease progression (Jung et al., 2006). In this study, the COMP levels in the synovial membrane were elevated in the early OA stages across all MIA doses; however, on day 28, upregulation was observed only in the 2 and 3 mg MIA groups (3- and 4-fold vs. the control group). In the synovial fluid, COMP levels were increased at the end of the experimental period (day 28 in all MIA doses).

In addition to inflammation-related factors, four MMPs were further investigated in this study. MMPs and their regulators (tissue inhibitors of metalloproteinases; TIMPs) are considered biological markers of OA (DeGroot et al., 2002; Rousseau and Garnero, 2012). Here, the levels of MMP-2, -3, -9, and -13 were determined. The gelatinases MMP-2 and MMP-9 are important factors in the pathogenesis of several diseases, including cancer, liver fibrosis, cardiovascular diseases and rheumatoid arthritis (Kurzepa et al., 2014; Radenkovic et al., 2014; Zhou et al., 2014; Radosinska et al., 2017). They are also reportedly involved in inflammation and inflammatory cell migration (Cui et al., 2017; Hannocks et al., 2019; Kim et al., 2012). Gelatinases also play an important role in the inflammatory response during the course of OA. In synoviocyte and anterior cruciate ligament fibroblast cell cultures, TNFα stimulation increases MMP-2 and MMP-3 levels (Wang et al., 2019). In rheumatoid arthritis (RA) synovial fibroblasts, MMP-2 and MMP-9 contribute to cell survival, proliferation and migration. MMP-9 has been shown to stimulate RA synovial fibroblast-mediated inflammation, whereas MMP-2 exhibited the opposite effect (Xue et al., 2014). Moreover, MMP-9 expression and activation are reportedly increased in septic native knee arthritis patients in comparison to aseptic knee arthritis patients (Fotopoulos et al., 2012). In cartilage and synovial fluid of OA patients, gelatinase protein levels are increased (Alunno et al., 2017; Duerr et al., 2004; Kim et al., 2011; Lipari and Gerbino, 2013); however, there may be differences between Asian and Caucasian patients (Zeng et al., 2015). In the present study, the gelatinase MMP-2 showed a gradual dose- and time-dependent increase in both gene expression and protein production. A significant increase was observed in the advanced phases of the disease at all MIA doses (from day 7 in qPCR and from day 21 in Western blot). This indicates a role for MMP-2 in joint tissue degeneration and inflammation in the later stages of OA in a rat model. As described above, early inflammation connected to MIA i.a. injection may explain the early increase in MMP-9 protein secretion by synoviocytes into the synovial fluid observed in almost all experimental groups in the current study. A similar increase at the end of the experiment, observed in the 3 mg MIA dose group, may indicate the role of MMP-9 in ECM degradation in the advanced stages of the disease in the highest MIA dose group, in which the OA lesions are the most severe. Lower MIA doses (1 and 2 mg) may not be sufficient to trigger such an effect in a 4-week period. However, it remains possible that lower MIA doses could trigger such an effect if the experimental period were longer. Further experiments are needed to clarify this effect, although most studies report an experimental period of no longer than 4 weeks. Regarding the gene expression levels of *Mmp-9*, we observed that synovial membrane samples provided a more substantial response than cartilage samples. The synovial membrane is a more secretory tissue compared to cartilage and is responsible for synovial fluid production, joint lubrication and maintaining homeostasis of the joint (Orr et al., 2017). In turn, cartilage does not play a primary secretory role in the joint and in fact degenerates over the course of the disease, further reducing its reactivity (Bryk et al., 2020).

MMP-3 (stromelysin 1) is also an important factor in osteoarthritis pathogenesis. In human synovial membrane cell culture and TNF-α-stimulated human cartilage experiments, MMP-3 levels are reportedly elevated (Sun et al., 2014). Additionally, in RA patients, MMP-3 serum levels are upregulated (Mahmoud et al., 2005) and can be reduced following anti-inflammatory (anti-TNFα) treatment (Sun et al., 2014). In OA patients, an elevated level of MMP-3 was also shown (Li et al., 2012). Moreover, the serum level of MMP-3 was higher in patients with OA changes in two or more locations (hands, hips, knees, spine, feet) than in people with only one location (knee joints) affected (Georgiev et al., 2018). In a reversible osteoarthritis rabbit model, MMP-3 and COMP levels were elevated in serum and synovial fluid samples from OA animals, which was correlated with OA severity (Chu et al., 2015). MMP-3 levels are also correlated with leptin concentrations in the synovial fluid of OA patients (Koskinen et al., 2011). In our study, at a low MIA dose (1 mg), MMP-3 gene expression and protein production increased in the initial stages of the experiment. At higher MIA doses (2 and 3 mg), we observed either late or constant upregulation of MMP-3 at both the gene and protein levels. This result may indicate an important role for MMP-3 in advanced OA (in the later experimental days at higher MIA doses). At the low MIA dose (1 mg), MMP-3 might be involved in the early inflammatory state; however, the OA grade in this group was lower than that in the 2 or 3 mg MIA groups; therefore, the contribution of MMP-3 to OA development may not be significant.

The final investigated matrix metalloproteinase, collagenase MMP-13, is an enzyme that plays a pivotal role in OA progression (Neuhold et al., 2001). Its knockout in mice results in deceleration of OA progression (Wang et al., 2013) and reduction of arthritis-evoked inflammation and cartilage erosion (Singh et al., 2013). In experimental equine OA, MMP-13 was elevated in synovial fluid samples (Ma et al., 2017). MMP-13 inhibition reduces cartilage erosion in animal models of RA (Jüngel et al., 2010) and blocks type II collagen degradation in bovine explants and human OA cartilage (Piecha et al., 2010). Moreover, in the synovial fluid of OA and RA patients, the level of MMP-13 is reportedly increased (Andereya et al., 2006; Kim et al., 2011; Özler et al., 2016). Li et al. demonstrated that in human OA cartilage, MMP-13 is elevated and suppresses cell proliferation (Li et al., 2019). MMP-13 also promotes cartilage degeneration via histone deacetylase (HDAC), and HDAC7 inhibition diminishes MMP-13 expression in chondrocytes (Higashiyama et al., 2010). MMP-13 inhibition may therefore also show therapeutic potential for OA treatment (Li et al., 2011). In the current study, MMP-13 protein levels in synovial fluid were elevated throughout the entire experimental period in all groups. In joint tissues, an early increase was observed at a lower MIA dose, whereas 3 mg MIA treatment resulted in MMP-13 elevation in cartilage samples in the later OA stages. *Mmp-13* gene expression was more elevated in synovial membrane samples than in cartilage, with a similar pattern of changes (an early increase at lower MIA doses and a prolonged increase at the highest dose). As noted above, the synovial membrane plays the primary secretory role in the joint and is responsible for synovial fluid production. This may explain the more significant effects observed in the synovial membrane than cartilage in *Mmp-13* gene expression, as well as its protein abundance in synovial fluid. Our results suggest that MMP-13 plays an important role in OA development, since its protein level (functionally more important than gene expression changes) was elevated across all groups. In the 3 mg MIA group, MMP-13 levels were also increased in cartilage at the later stages of OA, indicating that the highest MIA dose causes severe OA-like lesions in a rat model.

It should be noted that OA triggers not only local changes but also broader changes in the nervous system (Murphy et al., 2012; Clauw and Hassett, 2017). Thakur et al. investigated the influence of 1 or 2 mg MIA treatment on changes in dorsal root ganglion cells in OA animals, demonstrating that ATF-3 (a sensitive marker of peripheral neuron stress/injury) signaling was increased in 2 mg MIA-treated animals than in a 1 mg MIA treatment group. 2 mg MIA injection also reduced intraepidermal nerve fiber density in plantar hind paw skin and produced spinal cord dorsal and ventral horn microgliosis, which was not observed in a 1 mg MIA group (Thakur et al., 2012). These data are consistent with our results and suggest that 2 mg MIA, in addition to cartilage degeneration, evokes significant biochemical changes not only locally in the joint but also in the nervous system, although 1 mg MIA is not sufficient to trigger such changes.

## Conclusion

In conclusion, at the behavioral level, both 1 and 3 mg MIA treatment triggered similar effects. However, at the biochemical level, 2 and 3 mg MIA treatment showed comparable effects, while 1 mg appeared insufficient to trigger substantive OA-like changes at the molecular level in a rat OA model. In turn, 3 mg MIA treatment provided the most severe response with respect to both inflammatory factor and MMP release. Although many global investigators use an MIA animal model to study the therapeutic potential of various compounds, the MIA-induced OA model in rats has not previously been fully described in the literature. Our results indicate that 2 mg MIA may represent the “gold standard” treatment, as the lowest possible dose that effectively triggers OA but does not induce overly high inflammation. Our study fills in critical missing information regarding OA development and progression in a rat model.

## Data Availability

The raw data supporting the conclusions of this article will be made available by the authors, without undue reservation.
