# Supplementary material for: Sodium Monoiodoacetate Dose-Dependent Changes in Matrix Metalloproteinases and Inflammatory Components as Prognostic Factors for the Progression of Osteoarthritis
Source: Front Pharmacol. 2021 Apr 28;12:643605. doi: 10.3389/fphar.2021.643605 (PMC8113822; doi:10.3389/fphar.2021.643605)
Supplement: Supplementary file 1 [file DataSheet1.docx]

Supplementary Material

# Supplementary Data

| **Tissue** | **Gene** | **MIA [mg]** | **Days post-MIA injection** | | | | |
| --- | --- | --- | --- | --- | --- | --- | --- |
|  |  |  | **Ctrl** | **2** | **7** | **21** | **28** |
| Synovial membrane | *Ccl2* | 1 | 1,0 ± 0,1 | **27,1*** ± 6,6** | 2,9 ± 0,4 | 1,1 ± 0,3 | 2,8 ± 0,3 |
|  |  | 2 |  | **20,1** ± 7,4** | 6,3 ± 0,7 | 7,6 ± 3,7 | 1,9 ± 0,4 |
|  |  | 3 |  | **25,3*** ± 2,7** | 3,5 ± 0,5 | **6,2* ± 0,7** | 4,2 ± 0,5 |
|  | *Cxcl1* | 1 | 1,0 ± 0,1 | **16,9*** ± 4,8** | 2,8 ± 0,7 | 0,9 ± 0,1 | 1,8 ± 0,4 |
|  |  | 2 |  | **23,0*** ± 1,1** | **3,7** ± 0,2** | 1,1 ± 0,3 | 1,6 ± 0,5 |
|  |  | 3 |  | **49,0*** ± 8,9** | 2,8 ± 0,5 | 1,7 ± 0,2 | 4,6 ± 1,5 |
|  | *Il-6* | 1 | 1,0 ± 0,2 | **44,3*** ± 6,2** | 1,0 ± 0,2 | 1,3 ± 0,2 | 0,8 ± 0,4 |
|  |  | 2 |  | **37,1*** ± 1,3** | 5,2 ± 2,5 | 4,6 ± 3,5 | 1,2 ± 0,4 |
|  |  | 3 |  | **23,0*** ± 6,0** | 1,9 ± 0,2 | 1,7 ± 0,8 | 6,8 ± 2,4 |
| Cartilage | *Comp* | 1 | 1,1 ± 0,2 | **0,1** ± 0,0** | **0,4** ± 0,1** | 0,6 ± 0,1 | 1,5 ± 0,3 |
|  |  | 2 |  | 0,1 ± 0,0 | 0,4 ± 0,2 | **2,6* ± 0,8** | 1,1 ± 0,3 |
|  |  | 3 |  | 0,1 ± 0,1 | 0,1 ± 0,0 | **2,5** ± 0,7** | 1,8 ± 0,2 |

**Supplementary Table 1.** Transcript abundance levels of selected genes in synovial membrane and cartilage samples of osteoarthritic rats. The results were assessed by quantitative polymerase chain reaction (qPCR). Total RNA samples were collected 2, 7, 21 or 28 days after OA induction. The results are presented as the mean group fold change ± SEM in comparison to the control group (healthy animals), n = 3-6 samples per group. Data were analyzed with one-way ANOVA followed by Dunnett’s post-hoc test. * denotes p < 0.05; ** denotes p < 0.01; *** denotes p < 0.001 vs. intact animals.

| **Antibody** | **Provider** | **Catalog no** | **Dilution** |
| --- | --- | --- | --- |
| Anti – CCL2 | Abcam | ab21396 | 1:1000 |
| Anti – COMP | Abcam | ab128893 | 1:1000 |
| Anti – CXCL1 | Abcam | ab86436 | 1:500 |
| Anti – IL1β | Abcam | ab9787 | 1:1000 |
| Anti – MMP2 | Abcam | ab2462 | 1:1000 |
| Anti – MMP3 | Abcam | ab52915 | 1:1000 |
| Anti – MMP9 | Abcam | ab38898 | 1:1000 |
| Anti – MMP13 | Abcam | ab39012 | 1:1000 |
| Anti – β actin | Sigma | A2228 | 1:15000 |
| Anti – Mouse | Vector Laboratories | BA-2020 | 1:4000 |
| Anti – Rabbit | Vector Laboratories | BA-1000 | 1:4000 |
| Anti – Armenian Hamster | Santa Cruz | sc-2789 | 1:2000 |

**Supplementary Table 2.** Primary antibodies used in Western blot assays.

| **Gene** | **Assay ID** |
| --- | --- |
| *B2m* | Rn00560865_m1 |
| *Mmp2* | Rn01538170_m1 |
| *Mmp3* | Rn00591740_m1 |
| *Mmp9* | Rn00579162_m1 |
| *Mmp13* | Rn01448194_m1 |
| *Ccl2* | Rn00580555_m1 |
| *Cxcl1* | Rn00578225_m1 |
| *Il-6* | Rn01410330_m1 |

**Supplementary Table 3.** TaqMan assays used for qPCR analysis.
